# Rat models of frozen shoulder: Classification and evaluation

**DOI:** 10.1002/ame2.12516

**Published:** 2024-12-03

**Authors:** Hezirui Gu, Wenqing Xie, Hengzhen Li, Shuguang Liu, Yusheng Li

**Affiliations:** ^1^ Department of Orthopedics, Xiangya Hospital Central South University Changsha China; ^2^ Xiangya School of Medicine Central South University Changsha China; ^3^ National Clinical Research Center for Geriatric Disorders, Xiangya Hospital Central South University Changsha China; ^4^ Department of Joint Surgery, Honghui Hospital Xi'an Jiaotong University Xi'an China

**Keywords:** endocrine modeling, injection, rat model, surgical internal immobilization

## Abstract

Frozen shoulder (FS), also known as adhesive capsulitis, is a condition that causes contraction and stiffness of the shoulder joint capsule. The main symptoms are persistent shoulder pain and a limited range of motion in all directions. These symptoms and poor prognosis affect people's physical health and quality of life. Currently, the specific mechanisms of FS remain unclear, and there is variability in treatment methods and their efficacy. Additionally, the early symptoms of FS are difficult to distinguish from those of other shoulder diseases, complicating early diagnosis and treatment. Therefore, it is necessary to develop and utilize animal models to understand the pathogenesis of FS and to explore treatment strategies, providing insights into the prevention and treatment of human FS. This paper reviews the rat models available for FS research, including external immobilization models, surgical internal immobilization models, injection modeling models, and endocrine modeling models. It introduces the basic procedures for these models and compares and analyzes the advantages, disadvantages, and applicability of each modeling method. Finally, our paper summarizes the common methods for evaluating FS rat models.

## Graphical abstract

Procedure for Surgical Internal Immobilization Modeling. After intraperitoneal anesthesia, the rat was placed on the operating table (A). In the lateral decubitus position, a longitudinal skin incision of approximately 3 cm was made along the inferior edge of the scapula, perpendicular to the spine of the scapula (B). After exposing the scapula and humerus (C), two small holes were made in the inferior angle of the scapula using a 23G needle (D). Two 2–0 sutures were then passed through the holes and around the humeral shaft, securing them tightly to fix the shoulder joint (E–H). All wounds were closed with interrupted 3–0 nylon sutures.

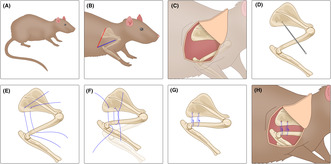

## 1 INTRODUCTION

Frozen shoulder (FS), also known as adhesive capsulitis, manifests as pain around the shoulder joint, with a progressive decrease in both active and passive range of motion (ROM) in all directions, leading to limited mobility.^1^, ^2^, ^3^ The main characteristics of FS are fibrotic hyperplasia of the fibrous tissue, accompanied by inflammation, neovascularization, and new nerve supply, which lead to capsular fibrosis and joint stiffness in the shoulder.^3^, ^4^, ^5^, ^6^, ^7^, ^8^ Epidemiological data indicate that the incidence of FS is about 2%–5%, mostly affecting middle‐aged and elderly people about the age of 50 years. The incidence is higher in women than in men, and most patients suffer from unilateral shoulder involvement.^9^, ^10^, ^11^ Based on the cause of the disease, FS can be classified into primary and secondary types.^1^, ^12^, ^13^ Immobilization, external trauma, diabetes, cardiovascular diseases, and thyroid diseases are usually considered possible triggers for secondary FS, with diabetes being particularly closely related to FS.^13^, ^14^, ^15^, ^16^ Also, recent clinical studies suggest that hyperlipidemic patients have a higher risk of developing adhesive capsulitis than healthy people.^17^, ^18^ In addition, it is reported in the relevant studies that calcific tendinitis (CT) has been confirmed as a major cause of secondary adhesive capsulitis. Studies have indicated that inflammation of the shoulder joint capsule and shoulder immobilization are potential triggers for adhesive capsulitis in patients with CT.^19^, ^20^ Both conditions exhibit some similarities in their pathological changes. Meanwhile, the application of ultrasound technology can facilitate the differential diagnosis and combined treatment of these two conditions.^19^, ^20^ Among patients with FS, about 35% have more than three comorbidities.^13^ However, even though the clinical characteristics and risk factors of FS are relatively clear, the pathophysiological mechanisms of the disease remain unclear, and therefore, there is no universally accepted optimal treatment method.^21^, ^22^, ^23^, ^24^ Currently, treatment methods can be broadly divided into surgical and nonsurgical categories.^25^ Additionally, although FS is considered a self‐limiting disease (with a recovery period of ~2 years), various studies have shown that symptoms such as shoulder stiffness, pain, and reduced mobility persist in some patients, affecting their quality of life.^26^, ^27^, ^28^

In current clinical studies, the progression of FS is typically divided into three stages, each characterized by specific histological changes.^29^ In 2010, Neviaser and Hannafin revised the original four‐stage progression of FS into three stages.^30^ The first stage of FS, also known as the freezing stage, is primarily characterized by pain without significant motion limitation. Histological examination during this stage reveals inflammatory responses in the fibrous synovium, vascular proliferation, and infiltration of inflammatory cells. This stage lasts ~3–6 months. The second stage of FS, known as the frozen stage, is marked by more severe and persistent pain, along with progressive limitations in shoulder joint motion, including internal rotation and abduction. During this period, histological changes include the disappearance of the axillary recess, synovitis, and capsular contracture in the shoulder. In the third stage of FS, known as the thawing stage, pain decreases and mobility gradually improves, although the degree and duration of improvement remain controversial.^1^, ^30^, ^31^, ^32^, ^33^ Nevertheless, there are no clear standards to distinguish these stages, and the progression of FS is continuous. Additionally, during the different stages of FS, histological changes such as thickening of the joint capsule and ligaments,^34^, ^35^, ^36^ joint adhesions,^37^ and type III collagen deposition^4^, ^38^ have been observed. However, it is still unclear which specific pathological change primarily causes capsular contracture, leading to pain and reduced mobility. It is well known that the treatment and rehabilitation of FS is a long and challenging process. Using human patients with FS directly as research subjects poses serious ethical issues. Related therapies must be proven completely safe in experimental animals before being applied to humans. Currently, many animal models of FS have been established. Common experimental animals such as mice, rats, dogs, and rabbits not only share genetic similarities with humans but also exhibit similar patterns of life activities and potential diseases.^39^ Therefore, these animal models can help us better understand and investigate the pathophysiological mechanisms and potential treatments of human FS. Of course, the anatomical structures of animals cannot be completely identical to those of humans. Therefore, choosing the appropriate animal model is crucial to better simulate the pathogenesis and histological changes in human FS. In this paper, we review the different rat models of FS, summarize the research progress on the pathogenesis and histological changes of FS, and outline potentially effective treatments. Additionally, we assess the advantages and disadvantages of each modeling method and determine whether they accurately simulate the pathophysiological characteristics of human FS, evaluating their specific roles in experiments.

## 2 THE IMPORTANCE OF RAT MODEL OF FS

**FIGURE 1:**
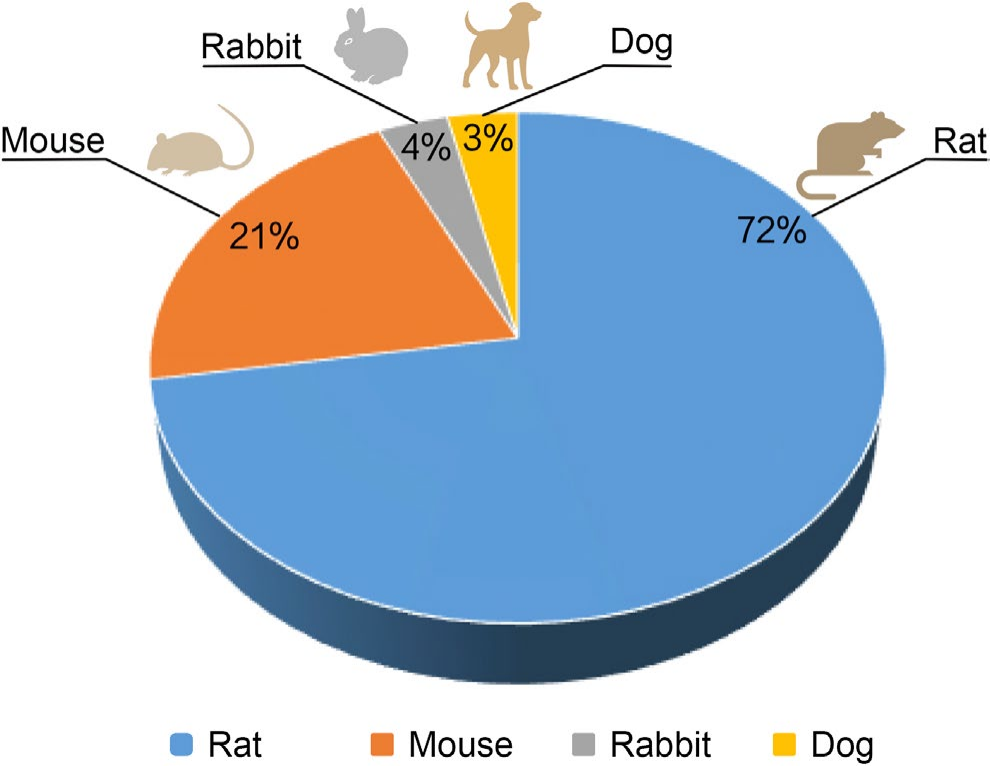
Animal species used in frozen shoulder animal models.

To thoroughly investigate the pathogenesis, pathological changes, and treatment efficacy of FS, scientists need to establish reliable animal models that simulate the disease as it occurs in humans. Among the various animal models available, species such as rats, mice, dogs, and rabbits are considered for modeling FS. Each animal has its unique advantages and limitations, but researchers must consider multiple factors when selecting the optimal model. Rats are often the preferred animals for modeling FS due to several significant advantages (Figure 1).

First, and most important, the skeletal and muscular structure of the rat's rotator cuff is very similar to that of humans. Studies have shown that, like humans, rats have a well‐developed supraspinatus tendon near the humerus and an acromion adjacent to the supraspinatus tendon. Additionally, the rat's supraspinatus tendon passes through an arch formed by the coracoacromial ligament, a structure not found in other animals with such high similarity to humans.^40^, ^41^ Second, rats have strong survival capabilities, are easy to maintain and breed, and have lower modeling costs and shorter cycles compared to larger animals. Additionally, rats have strong resistance to infections, effectively preventing complications during modeling that could affect experimental results, thereby increasing the success rate and accuracy of experiments. Animal models of FS should involve two important characteristics of the disease: capsular contracture of the shoulder joint and long‐term reduction in shoulder mobility,^42^ both of which are well represented in rat models of FS. In conclusion, based on the anatomical and physiological similarities, research background, ease of experimental manipulation, lifecycle characteristics, and economic benefits, rats have become the preferred choice for establishing animal models of FS. This helps researchers to systematically understand the complexity of FS and develop more effective treatment strategies.

## 3 CLASSIFICATION OF RAT MODELS OF FS

### 3.1 External immobilization models

Immobilization is an important inducing factor of secondary FS^1^, ^5^, ^43^; thus external immobilization rat models have been widely used in FS research. Typically, molded plaster or bandages are used to immobilize the shoulder of experimental rats to simulate the immobilization process of human FS. In previous studies using Sprague–Dawley (SD) rats, Liu et al.^44^ immobilized the rats' shoulders and thoraxes with molded plaster for 1, 2, 3, and 4 weeks, with the shoulder joint internally rotated at 90°. Histological and pathophysiological changes in the rat shoulder were observed at each time point. The rats were able to walk, eat normally, and did not develop complications after immobilization. They observed synovial and capillary proliferation in the early stages of immobilization (2 or 3 weeks). In the middle to late stages (3 or 4 weeks), subscapular adhesions were observed, along with increased accumulation of type I and III collagen in the synovial intima. These results are consistent with previous human FS studies,^4^, ^37^, ^45^ confirming the feasibility of the plaster immobilization rat model. Kim et al.^46^ improved upon previous experimental methods by still using molded plaster to immobilize the shoulders and thoraxes of SD rats, positioning the shoulders in adduction and the elbows in flexion, and observing at 3 days, and 1, 2, 3, 4, 5, and 6 weeks. They found that the shoulder abduction angle of the experimental rats significantly decreased after just 1 week of immobilization. Histologically, inflammatory cell infiltration, synovial hyperplasia, and capsular thickening appeared after just 3 days and progressively worsened until 3 weeks. After 3 weeks of immobilization, the inflammatory cells decreased, and the primary symptom became synovial fibrosis. Consequently, the researchers concluded that this plaster immobilization model effectively simulated the inflammation‐induced fibrosis observed in human secondary FS^3^, ^6^, ^47^, ^48^ and determined that the model could be completed within 3 weeks of immobilization. Kim et al. not only successfully established a rat model of FS but also conducted a rigorous evaluation,^46^ providing guidance for future modeling and model assessments.

Many experiments directly use or modify Kim's plaster immobilization method. For example, in a study on the treatment of FS,^49^ researchers conducted histological studies on days 3, 7, and 21 of immobilization. They found overexpression of three inflammatory cytokines in rats, which was consistent with the results of comparing primary FS patients to normal individuals. The experiment not only demonstrated that matrix metalloproteinases (MMP) are involved in the pathogenesis of FS but also showed that the plaster immobilization model in rats exhibits certain histological similarities to primary FS in humans,^50^ indicating its high utility. In another study, Taguchi et al.^51^ performed angiographic examinations on the immobilized group of rats and found abnormal vascular proliferation in their shoulders. This result is consistent with Okuno et al.'s findings of abnormal angiographic staining in the shoulders of human FS patients,^52^ further indicating that the plaster immobilization model can accurately simulate the pathological process of human FS. The aforementioned studies indicate that using plaster for external immobilization modeling is reliable, and the model can be completed in ~3 weeks. Performing immobilization externally is straightforward, consumes less time and is inexpensive, and effectively avoids complications or subsequent impacts caused by surgery. However, the plaster immobilization model still has disadvantages. During the immobilization process, rats may damage the immobilization device due to discomfort, affecting the progress and results of the experiment. Additionally, differences in limb positions during immobilization may cause errors.^53^ Currently, plaster modeling is widely used in studies on the pathogenesis of FS, particularly in researching the causes of synovial fibrosis, playing an important role. Relevant studies on rat plaster external immobilization modeling for FS are summarized in Table 1.

**TABLE 1:** Studies of external immobilization models.

Rat age	Modeling time	Key findings	References
8 months	4 weeks	Immobilization‐induced subscapular bursa (SSB) adhesion and collagen deposition may contribute to the development of frozen shoulder	[44]
7 weeks	6 weeks	This experiment demonstrated that a rat shoulder contracture model induced by plaster immobilization can produce a pathological process similar to human shoulder periarthritis fibrosis, with the model being established in 3 weeks	[46]
7 weeks	4 weeks	The modeling demonstrated that specific MMPs play an important role in the process of shoulder periarthritis in both humans and rats	[49]
7 weeks	6 weeks	The model successfully simulated angiogenesis observed in human shoulder periarthritis patients and suggested that transcatheter arterial embolization can effectively alleviate symptoms of shoulder periarthritis in rats	[51]
12 weeks	3 weeks	The model demonstrated that salvianolic acid B can alleviate inflammation and inhibit CD36‐mediated pathological fibrosis in frozen shoulder	[54]
7 weeks	3 weeks	The model demonstrated that intra‐articular injection of triamcinolone can reverse the structural and functional changes caused by frozen shoulder in rats	[55]
7 weeks	3 weeks	The experiment proved the effectiveness of the plaster immobilization model and demonstrated that massage and oral dexamethasone can effectively maintain the shoulder structure and function of rats with frozen shoulder	[56]
7 weeks	3 weeks	The study identified a pathway in the mRNA of rat bone marrow mesenchymal stem cells that alleviates symptoms of frozen shoulder in rats, providing a possible approach for future treatment of frozen shoulder in humans	[57]
8 weeks	3 weeks	This study developed an injectable hyaluronic acid hydrogel and demonstrated its ability to alleviate shoulder inflammation and fibrosis through a rat frozen shoulder model	[58]

Abbreviations: MMP, matrix metalloproteinase; mRNA, messenger RNA.

### 3.2 Surgical internal immobilization models

Besides immobilization, external trauma (e.g., surgery) is a major inducer of secondary FS.^26^ In experimental studies, surgical methods are often used to fix the scapula of the rat to the ipsilateral upper limb to simulate the restricted shoulder movement seen in human FS patients. Common immobilization and suturing materials include metal wires, fiber sutures, plastic plates, and steel plates. Kanno et al.^53^ found that plaster immobilization might have errors due to different limb positions in each experimental animal, so they chose to use internal immobilization to establish a rat shoulder contracture model. Researchers made surgical incisions at the humerus and scapula, inserted a plastic plate at each incision, and fixed them with flexible steel wires. Additionally, two steel plates were bound to the scapula with metal wires and steel nails to achieve rigid‐shoulder immobilization. It is noteworthy that Kanno et al. set the internal immobilization angle at 60° abduction, which precisely simulates 0° abduction in humans. The rats survived normally after surgery, and histological examinations and rotational angle measurements were conducted 8 weeks postmodeling. They found that compared to the control sham surgery group, the abduction and rotation angles in the modeling group were both reduced, consistent with previous research results,^37^ indicating the successful establishment of the shoulder contracture model. Histologically, they observed synovial adhesions in the surgical group and a significant shortening of the intimal length, similar to the causes of contracture reported by Trudel et al. in previous studies on knee joint immobilization in rats.^59^, ^60^ However, no inflammation was observed in the shoulders of the rats in the modeling group, which seems different from the pathogenesis of human FS.^3^, ^31^, ^61^ Therefore, the reliability of this model still requires further validation.

**FIGURE 2:**
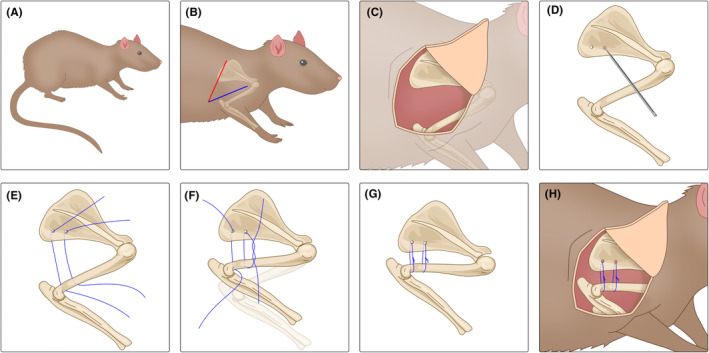
Procedure for surgical internal immobilization modeling. (A) After intraperitoneal anesthesia, the rat was placed on the operating table. (B) In the lateral decubitus position, a longitudinal skin incision of ~3 cm was made along the inferior edge of the scapula, perpendicular to the spine of the scapula. After the (C) scapula and humerus were exposed, (D) two small holes were made in the inferior angle of the scapula using a 23G needle. (E–H) Two 2–0 sutures were then passed through the holes and around the humeral shaft, securing them tightly to fix the shoulder joint. All wounds were closed with interrupted 3–0 nylon sutures.

Okajima et al.^62^ also used surgical methods to establish an internal immobilization contracture model but simplified the immobilization process. Like Kanno, they made incisions at the shoulder joint but did not use steel plates or plastic plates. Instead, they used two polyester fiber sutures to pass through the humerus and scapula, which were then tightened to fix the glenohumeral joint. The immobilization devices were removed after 8 weeks of modeling, and the ROM of the rats' glenohumeral joints was recorded. Researchers found that the total ROM decreased by approximately 63% immediately after the immobilization ended, and there was still a 19% reduction in ROM 8 weeks after the immobilization ended, with no further improvement. This is consistent with previous reports on human FS, indicating that the movement restriction caused by FS can naturally lessen but cannot fully recover.^26^, ^27^, ^28^ Histologically, capsular thickening and partial adhesions were observed. These experimental results indicate the feasibility of this internal immobilization contracture model in simulating human FS. Additionally, studies by Ochiai et al.^63^ and Villa et al.^42^ have demonstrated that bone‐external sutures can be used to establish contracture models, although the immobilization materials they used differed: Ochiai et al. used 2–0 FiberWire, whereas Villa et al. used braided polyester sutures. Currently, there is no definitive standard for the superiority of these immobilization materials, but the observed results in experimental rats, such as reduced shoulder ROM, increased stiffness, and decreased rotation angles, all demonstrate the reliability of internal immobilization shoulder contracture models. These models have been widely modified and applied in FS research. The specific procedure for surgical internal immobilization modeling is shown in Figure 2.

Overall, the surgical internal immobilization model has been proven by many experiments to be a reliable animal model for FS. Modeling can be completed in ~8 weeks, consistently inducing shoulder contracture, capsular thickening, and fibrosis in rats to simulate the pathological process of human FS. However, this model also has certain disadvantages. The surgical modeling and subsequent suturing process may lead to complications, affecting the normal life activities of the rats and potentially resulting in insufficient sample support for experimental results. Additionally, anatomical differences between humans and rats limit the applicability of surgical modeling.^40^ Currently, the surgical internal immobilization model is often used in the research and development of new therapies for FS, playing an irreplaceable role. Related studies on surgical internal immobilization modeling of FS in rats are summarized in Table 2.

**TABLE 2:** Studies of surgical internal immobilization models.

Rat age	Modeling time	Surgical materials	Key finding	References
6–8 weeks	8 weeks	Number 2–0 braided polyester suture	By measuring shoulder joint stiffness and range of motion, this study demonstrated the effectiveness of using internal immobilization to establish a rat model of frozen shoulder and hypothesized that it may be caused by changes in the synovial capsule	[42]
12 weeks	8 weeks	Steel plates, plastic plates, screws, and flexible metal wires	The experiment confirmed the successful establishment of the model through a joint range of motion measurements and histological examinations, providing valuable insights into future internal immobilization modeling	[53]
6–8 weeks	8 weeks	Number 2–0 FiberWire	The experimental results suggest that the upregulation of capsular calcitonin gene‐related peptide (CGRP) is closely associated with the pain observed in frozen shoulder	[63]
12 weeks	8 weeks	Steel plates, plastic plates, screws, and flexible metal wires	In the experimental model, silencing the *Smad4* gene was able to inhibit synovial tissue fibrosis, having a positive effect on the treatment of frozen shoulder	[64]
3 months	8 weeks	Number 1–0 FiberWire	Animal experiments confirmed that oral vitamin C can reduce the thickening of the axillary recess in rats with frozen shoulder, suggesting that vitamin C may be used for noninvasive treatment of frozen shoulder	[65]
12 weeks	8 weeks	Number 2–0 FiberWire, using 5–0 Vicryl for the subcutis and 4–0 Maxon for intracutaneous suture	According to the model study results, platelet‐rich plasma can prevent significant structural changes in the posterior synovium of rats and does not present any clinical side effects	[66]
6–8 weeks	8 weeks	Number 2–0 sutures, all wounds were closed using 3–0 nylon	In this animal experiment, TET (Tetrandrine) was effective in preventing the occurrence and progression of frozen shoulder and alleviating symptoms at all stages of the condition	[67]
8 months	8 weeks	Number 2–0 braided polyester suture(TI‐CRON)	This experiment utilized an internal immobilization rat model of frozen shoulder to verify that intra‐articular corticosteroid injections are more effective than oral corticosteroids in improving shoulder mobility in rats	[68]

### 3.3 Other models

Beyond the two immobilization models previously mentioned, the authors have observed a distinctive method of injection modeling. In their experimental work, Chen et al.^69^ injected adenovirus‐TGF‐β1 into the shoulder capsules of rats under sterile conditions with ultrasound guidance. Five weeks postmodeling, the stiffness of the shoulder joints was assessed. The findings confirmed that rats injected with adenovirus‐TGF‐β1 exhibited typical signs of FS, likely because TGF‐β1, a cytokine known for its fibrotic and inflammatory properties, plays a critical role in the development of this condition.^69^, ^70^, ^71^ Then, the treated rats were used to demonstrate that PPAR‐γ agonists could effectively mitigate rat FS by reducing fibrosis.^69^ Although currently the application of injection modeling techniques is still in the experimental stage, they are simpler to implement compared to traditional immobilization methods, and they result in fewer complications and higher survival rates among the rats. Furthermore, ongoing research indicates a strong link between various cytokines, such as those from the MMP and interleukin (IL) families,^49^ and the inflammatory and fibrotic alterations in the shoulder capsule. Therefore, as research and experimental methods continue to advance, the use of injection modeling techniques is likely to become more widespread in the future.

In the development of models for studying FS, researchers have gone beyond traditional immobilization techniques to include other risk factors associated with the condition as seen in humans. These innovative modeling methods encompass environmental simulations such as models that replicate cold, damp conditions, and combinations of ice therapy with continuous mechanical injury. Additionally, models simulating endocrine system disorders, such as diabetes or thyroid diseases, are employed. Importantly, these diverse approaches to model construction have been primarily used by Chinese scholars who integrate principles of traditional Chinese medicine (TCM) into their methodologies. This integration of TCM theories with modern scientific research offers new perspectives and tools for a more comprehensive understanding and treatment of FS.

## 4 EVALUATION OF RAT MODELS OF FS

As noted earlier, a wide variety of rat models are available for studying FS, each with distinct advantages, disadvantages, and applicable scenarios. Studies referenced in Kim et al.^46^ and Kanno et al.^53^ have confirmed the feasibility of these modeling methods, leading many experiments to adopt these techniques without further evaluating the rat models. However, subtle variations in the experimental animals, immobilization techniques, and duration of modeling can introduce discrepancies in the outcomes, which may influence subsequent research. Therefore, it is recommended that rat models be evaluated after their development. In this section, the authors review common evaluation methods for rat models of FS, offering guidance and support for the development of future‐related models.

### 4.1 Shoulder joint mobility

Decreased shoulder mobility is the most characteristic symptom of FS and serves as a crucial diagnostic criterion.^72^, ^73^, ^74^, ^75^ Consequently, assessing changes in the mobility of the shoulder joint in rat models is essential for determining the success of the model. Commonly measured parameters include the ROM, abduction angle, and rotation angle of the shoulder. Researchers apply torque to the humerus and forearm to measure these angles in rats, noting that a significant reduction in ROM (about 60% compared to controls) validates the model.^42^, ^53^ Importantly, some studies have reported a partial rebound in ROM after immobilization is removed,^62^ which mirrors the clinical observations in human FS.^27^, ^28^, ^76^ However, a reduction in ROM alone cannot definitively distinguish FS from other rotator cuff disorders, necessitating the use of additional diagnostic criteria.^1^

### 4.2 Histological changes

Current research indicates that contraction of the shoulder joint capsule may lead to pain and mobility loss in patients.^77^, ^78^, ^79^, ^80^ In the modeling process, monitoring histological changes in experimental rats can confirm whether contraction of the shoulder joint capsule occurs, thereby assessing the model's reliability. Typically, researchers embed rat synovial specimens in paraffin and then stain them with specific agents such as hematoxylin and eosin for observation and measurement. Reports on human FS suggest that observable changes associated with capsular contraction include reductions in synovial length, adhesions, and synovial fibrosis,^73^, ^81^, ^82^ which can serve as benchmarks for validating modeling results. Similar histological changes have been noted in rat models; for instance, Liu et al.^44^ observed synovial hyperplasia and partial adhesions in the shoulder joints of rats during modeling. Kim et al.^46^ reported significant capsular thickening and synovial fibrosis, whereas Kanno et al.^53^ documented reductions in synovial length and partial adhesions, findings that closely resemble the pathological changes seen in human FS. Furthermore, traditional imaging methods such as arthrography, X‐ray, magnetic resonance imaging, and ultrasound are already employed in diagnosing FS in patients.^83^, ^84^ Imaging studies have revealed humeral atrophy, reduced capsular distention, obliteration of the axillary recess, and joint capsule thickening and edema,^35^, ^73^, ^85^ all indicative of capsular contraction.^86^ Thus, employing imaging techniques to observe capsular contraction in modeled rats is a vital method to verify the success of the model. Additionally, although a variety of histological changes occur in FS, no single “gold standard” exists that can directly differentiate FS from other shoulder disorders; evaluations should therefore be multifaceted.

### 4.3 Immunohistochemistry

Compared to the two previous assessment methods, immunohistochemistry is often the most neglected. In the context of FS, typical immunohistochemical markers include collagen and cytokine levels. Research indicates that changes in various types of collagen are closely linked to histological alterations in shoulder tissues, with excessive deposition of type III collagen potentially leading to stiffness and fibrosis of the shoulder joint capsule.^38^, ^47^, ^87^ Furthermore, specific cytokines and protein families have been identified as playing critical roles in the progression of FS. Elevated expressions of MMP family members,^49^, ^88^, ^89^ IL family cytokines,^49^, ^69^, ^90^ and TGF‐β^69^, ^90^, ^91^ in patients with FS, compared to healthy individuals, are thought to promote inflammatory responses and abnormal vascular proliferation, ultimately resulting in fibrosis and joint contraction.^4^, ^6^, ^32^, ^47^ During rat model development, both Liu et al.^44^ and Kanno et al.^53^ noted increased type III collagen in the shoulder joints during immobilization. Cho et al.^49^ found significant increases in MMP‐2, MMP‐9, and IL‐6 levels postmodeling via immunohistochemistry. Similarly, Ding et al.^64^ reported upregulated expressions of TGF‐β1 and MMPs in modeled rats. These findings underscore the importance of immunohistochemical analysis as a vital tool not only for evaluating modeling outcomes but also for providing insights into the development of new gene therapies in studies of human FS.^64^, ^92^, ^93^

## 5 PROSPECTS

The variety of risk factors associated with human FS suggests several directions for developing rat models of this condition. Notably, diabetes shows a significant correlation with FS in humans.^15^, ^94^, ^95^, ^96^ A meta‐analysis indicated that diabetic patients have a 30% likelihood of developing FS, which is five times higher than in nondiabetic controls.^97^ Additionally, diabetic patients are reported to have a poorer prognosis for FS and a higher tendency for bilateral involvement.^98^, ^99^ In one study, researchers examined changes in the ROM in the glenohumeral joint of diabetic rats. Inducing type 2 diabetes in SD rats with a high‐fructose diet^100^ showed that the ROM in all shoulder planes decreased by more than 15% in the diabetic group. Another study observed an increased incidence of synovitis in the joint capsules of diabetic rats. These results provide reliable theoretical support for establishing a diabetic rat model of FS. Furthermore, thyroid disorders are recognized as a trigger for FS.^101^, ^102^, ^103^ Studies have shown a significantly higher incidence of hypothyroidism among patients with FS compared to controls.^104^ With a robust theoretical basis for endocrine modeling already in place, further exploration and establishment of these models are both anticipated and promising for future research. Besides endocrine models, employing rat CT models is a potential future direction for FS modeling. Several studies have reported that CT and concurrent inflammatory responses and shoulder immobilization are potential risk factors for secondary adhesive capsulitis.^19^, ^20^ Currently, the technology for establishing a rat CT model through collagenase induction is relatively advanced.^105^, ^106^ Therefore, these theoretical and experimental foundations provide a solid basis for utilizing the CT model to develop the FS model, making this new modeling approach promising.

## 6 CONCLUSION

Rat models are indispensable in exploring the pathogenesis and optimal treatment strategies for human FS. These models involve methods such as external immobilization, surgical internal immobilization, environmental simulation, and endocrine modeling. Each method presents its own set of strengths and weaknesses, necessitating careful selection based on specific research requirements. Although progress has been made in the study of rat models for FS, notable discrepancies remain between these models and the actual conditions in humans. Furthermore, a standardized approach for evaluating the effectiveness of these models has not yet been established. Future research should prioritize the development of diverse rat models tailored to different research contexts and formulate standardized criteria for assessing the efficacy of these models. Our study summarizes the commonly used methods for inducing FS in rat models, clarifies the advantages and disadvantages of each method as well as their applicable scenarios, and proposes novel modeling approaches. This assists researchers in selecting the most appropriate modeling methods for further investigation into the pathophysiological mechanisms and optimal treatment method of human FS, thereby achieving more efficient outcomes. This also holds significant implications for clinical practice.

## AUTHOR CONTRIBUTIONS

**Hezirui Gu:** Writing – original draft. **Wenqing Xie:** Data curation; software; writing – review and editing. **Hengzhen Li:** Investigation; visualization. **Shuguang Liu:** Conceptualization; funding acquisition; supervision. **Yusheng Li:** Conceptualization; funding acquisition; resources; supervision.

## FUNDING INFORMATION

This work was supported by the National Key R&D Program of China (2021YFC2502100, 2023YFC3603404, 2019YFA0111900); the National Natural Science Foundation of China (82072506, 82272611, 92268115); the Hunan Provincial Science Fund for Distinguished Young Scholars (2024JJ2089); the Hunan Young Talents of Science and Technology (2021RC3025); the Provincial Clinical Medical Technology Innovation Project of Hunan (2023SK2024, 2020SK53709); the Provincial Natural Science Foundation of Hunan (2020JJ3060); the National Natural Science Foundation of Hunan Province (2023JJ30949); the National Clinical Research Center for Geriatric Disorders, Xiangya Hospital (2021KFJJ02, 2021LNJJ05); the Hunan Provincial Innovation Foundation for Postgraduate (CX20230308, CX20230312); and the Independent Exploration and Innovation Project for Postgraduate Students of Central South University (2024ZZTS0163).

## CONFLICT OF INTEREST STATEMENT

The authors declare that they have no known competing financial interests or personal relationships that could have appeared to influence the work reported in this paper.

## ETHICS STATEMENT

Not applicable.
